# Evaluation Of Atrial Fibrillation Burden Before Catheter Ablation Predicts Outcome After Pulmonary Vein Isolation

**Published:** 2009-05-15

**Authors:** Alexander Berkowitsch, Thomas Neumann, Malte Kuniss, Roland Brandt, Sergej Zaltsberg, Heinz F Pitschner

**Affiliations:** Dept. of Cardiology, Kerckhoff-Klinik, Bad Nauheim, Germany

**Keywords:** atrial fibrillation, atrial fibrillation burden, pulmonary vein isolation, catheter ablation, prediction, outcome

## Abstract

**Background:**

Paroxysmal atrial fibrillation (PAF) is defined as recurrent AF terminating spontaneously within 7 days. This definition allows the consideration of any AF occurrence lasting < 7 days as paroxysmal, irrespective of the frequency and duration of episodes. The aim of this study was to investigate symptomatic AF burden (AFB) defined as total duration of symptomatic AF episodes within 3 months prior to abalation, for prediction of outcome after pulmonary vein isolation (PVI).

**Methods:**

A total of 320 consecutive patients with symptomatic AF (PAF=244, men=214, age=58 y) were enrolled. AFB in patients with PAF was defined as time spent in AF within 3 months prior to PVI. After the AFB cut-off point was optimized at 500 h, patients with PAF were categorized into 2 groups: Group 1 - patients with AFB< 500 h (n=192), Group 2 - patients with AFB≥ 500 h (n=52). Patients with persistent AF (PersAF, n = 76) comprised control group (Group 3). PVI was performed either with irrigated tip catheter (n=215) or using cryoballoon (n=105). The endpoint of study was first documented recurrence of AF >30 sec.

**Results:**

Symptomatic AFB was found to be appropriate for prediction of outcome after PVI. The freedom from AF within 2 years was observed in 69%, 31%, and 43% patients in Group 1, 2 and 3, respectively (Group 1 vs. Group 2, p < .001; Group 1 vs. Group 3, p< .001; Group 2 vs. Group 3, p = 0.46).

**Conclusions:**

Low AFB < 500 h /3 months was associated with better outcome after PVI. Patients with PAF and high AFB should be treated as patients with PersAF.

## Introduction

 Pulmonary vein isolation (PVI) has been shown to be effective for treatment of paroxysmal atrial fibrillation (PAF) [1-3]. However, in up to 40 % of patients with PAF, a re-do procedure may be required [4]. Generally, the primary success rate of PVI is lower in patients with persistent AF compared with PAF [5]. Lower success rates have also been seen in patients with significant structural heart disease [6]. Other predictors of poor outcome include significant left atrial (LA) enlargement (> 55 mm) and advanced age (> 65 years) [7]. 

A recently published Expert Consensus Statement has recognized the significance of symptomatic status at enrollment for outcome after PVI [8]. The assessment of symptoms is particularly important in patients with PAF. According to the definition of HRS/EHRA/ECAS, PAF is defined as recurrent AF (> 2 episodes) that terminates spontaneously within 7 days [5,8]. This definition allows considering any AF lasting < 7 days as paroxysmal, irrespective of the frequency and duration of episodes within the observation period. The quantitative evaluation of symptomatic AF is possible by estimation of AF burden (AFB). The aim of this study was to assess the usefulness of symptomatic AFB, defined as total duration of AF episodes within 3 months prior to first ablation for AF, on maintenance of sinus rhythm during 2 years of follow-up (primary endpoint). The secondary endpoint was to identify the cut-off point for AFB in patients with paroxysmal AF allowing patients at high and low risk of AF recurrence within 2 years follow up to be discriminated between. The impact of short and long left atrial diameters evaluated in four-chamber echocardiography projection on outcome after PVI was also investigated in this study.

## Methods

### Baseline data

 A total of 320 consecutive patients with symptomatic AF refractory to beta-blockers and to ≥ 1 antiarrhythmic drug including class I agents, sotalol, and amiodarone, were enrolled into the study. The baseline characteristics of patients are shown in Table 1.

### Assessment of AF burden

 Three months prior to the procedure, patients with PAF were asked to keep a diary of their symptoms. AFB was calculated as total duration of AF episodes recorded in the diary as described in our previous study [9].  We decided to use this approach again because of the simplicity as well as reliability of evaluation and because other methods such as continuous 7-day Holter ECG monitoring provide limited information due to the short duration of the recording, whereas transtelephonic ECG monitoring is not widely applicable in routine clinical practice. Furthermore, in previously published studies the incidence of asymptomatic AF has been shown to increase up to 37% after ablation, but only 5% were fully asymptomatic before the procedure [10,11]. The last reason for using this approach was follows: we recognize that symptoms reported by patient cannot fully reflect real AF episodes. The important question is whether the residues between reported symptoms and actual number AF episodes which have occured are randomly distributed or have systematical pattern. In the first case the AF burden as calculated in our study is random number and subsequently can not be revealed as significant predictor in ROC analysis. In other case the residues are systemically distributed and the parameter can be used for diagnostics. In details this issue is described below in paragraph 'statistical analysis'.

### Ablation procedures

In 108 patients, 422 PVs were isolated using a 3.5 mm irrigated tip-catheter (5-mm Tip, Celsius Thermo-Cool™, Biosense Webster). The cut-off temperature of generator was 42” C and energy delivery was limited to a maximum of 35 W. Successful ablation was defined as a complete disappearance of the fragmented signals during mapping with a decapolar Lasso catheter (Lasso TM, Biosense Webster) at the PV antrum. If there were residual signals recorded after ablation, the site was paced to determine whether the far field signals were present.

 In other 107 patients treated with radiofrequency, mapping was performed using high density mapper (MESH. Bard inc. USA). We started an ipsilateral isolation of both sides. The complete loss of PV and antral atrial signals under the mapping electrodes was defined as complete antral PVI.

In the remaining 105 patients, 420 PVs were isolated using a 23 mm or 28 mm cryo balloon. If necessary, the residual gap was closed using the large tip catheter FreezorMax™. Mapping was performed by either a suitable Lasso catheter or a 3-D mapping system (NavX™). The balloons were advanced into the antrum. The balloon was inflated and the degree of occlusion was tested by contrast injection under fluoroscopic control. Ablations at a single site were repeated with guide wire positions in different branches of the PV to obtain better tissue contact in different parts of the antrum. If complete isolation with one balloon size could not be achieved, another balloon size and/or a larger tip were used to complete the PVI. The baseline data of patients according to ablation technique are shown in the Table 1.

In 244 patients considered according to the definition of HRS/EHRA/ECAS as having PAF only PVI was performed.  In remaining 76 patients with persistent AF, irrespective of the energy used for PVI, additional linear lesions such as a roof line and the mitral isthmus line were also performed using a radiofrequency catheter.

### Assessment of the left atrial size

**Figure 1:**
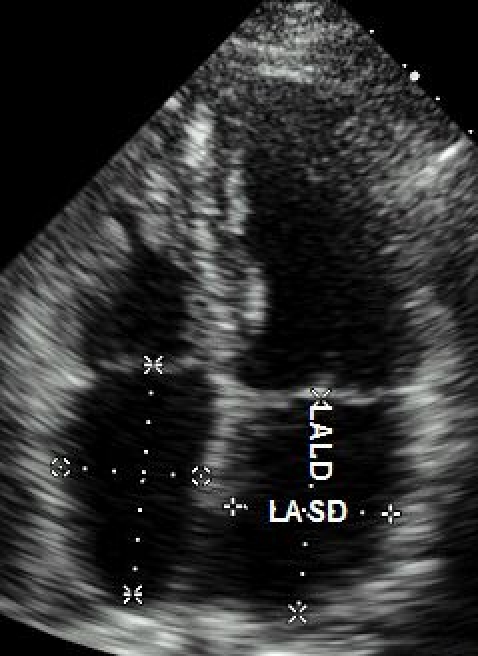
Measurement of left atrial size performed using apical four-chamber projection. LASD- left atrial short diameter; LALD-left atrial long diameter

Left atrial short and long diameters (LASD (parasternal measurement); LALD-apical measurement) were assessed in the apical four-chamber projection [12] (Figure 1).

### Follow up

All patients were seen in our outpatient clinic 3 months after catheter ablation and every 3 months thereafter for 2 years and additionally in the case of symptomatic recurrence of AF. A 7-day Holter ECG was performed at each follow-up visit. After discharge, patients treated with antiarrhythmic drugs prior to ablation, received the same antiarrhythmic drug therapy during the first 3 months. If sinus rhythm was maintained, antiarrhythmic therapy was terminated three months after PVI. Beta-blockers were continued in patients with hypertension or coronary artery disease. The endpoint of the study was the first documented AF recurrence > 30 s. The first three months after ablation were considered as a blanking period.

### Statistical analysis

This study was designed as a validation cohort study. Our aim was to test whether the AFB calculated based on patients' symptoms and left atrial diameters may be used for prediction of outcome and if yes to define and validate cut-off values for both parameters that would allow discriminating between patients with high and low risk of AF recurrence after ablation. To solve this issue ROC curve analysis should be performed [13].

In the case of absence of the gold standard or if independent data set is not available, the parameters of interests should be analyzed by randomization of study patients in two groups - one to derive the model and the other to validate it [13]. We randomly allocated 244 patients with PAF into two subsets of 122 each - the training set and the validating set. The data for patients randomized to training and validating sets are shown in the Table 2. The cut-off values for AFB and both diameters of the left atrium were estimated using maximum likelihood ratio (LR) derived from the ROC-curve analysis based on a training set. After the cut-off values were defined in the training set, they were tested in the validating set. Area under ROC curve, positive and negative predictive accuracy (PPA, NPA) of defined cut-offs were calculated.  After predictive accuracy of cut-off values was confirmed in the validating set, the patients were clustered together. Furthermore, the patients with PAF were categorized into 2 groups according to the defined cut-off point of AFB: Group 1 - patients with AFB< cut-off and Group 2 - patients with AFB≥ cut-off. The remaining 76 patients with persistent AF were considered as a control group (Group 3). The outcome was analyzed using Kaplan-Meier analysis and a multivariate Cox regression model with adjustment for all clinical variables included in baseline characteristics and ablation tools.

## Results

### Training set

Out of 122 patients with PAF randomized to training set 45 (36.9 %) reached endpoint. The test did not reveal an effect of the applied energy source on the outcome (Table 3). The area under the ROC-curve by AFB, left atrial long and short diameters  was .62, .60 and .53, respectively (Table 4). Subsequently, left atrial short diameter could not be used for prediction of outcome. Maximum likelihood ratio of AFB and left atrial long diameters was found at 500 hours (LR=2.33) and at 60 mm (LR=2.05), respectively. As shown in Table 4, positive and negative predictive accuracy of AFB by setting the cut-off point at 500 h was 58% and 69%, respectively. The predictive accuracy of left atrial long diameter with cut off point of 60 mm. was also moderate, PPA-54% and NPA-65%. However, both parameters allowed patients with high and low risk of AF recurrences after PVI to be discriminated between.

### Validating set

As in the training set the effect of the ablation energy could not be revealed (Table 3). The area under the ROC curve was .72 and .61 for AFB and LALD, respectively (Table 4). Similar to the training set maximum LR=6.05 and 2.24 were found at 500 hours and 60 mm, for AFB and LALD, respectively. The PPA was 81% and 61%, and the NPA was 70% and 64%, respectively (Table 4). Although the predictive accuracy of dichotomized AFB appeared to be stronger in the validating test, the performed z-test (z=1.50, p=.19) did not reveal statistical differences between areas under ROC curves. The difference between areas under ROC curves composed for LALD could also not be revealed (z=.47, p=.69). Therefore, we can use both of the tested cut-off values for the prediction of outcome in the whole group. 

### Impact of AFB and LALD on outcome after PVI

**Figure 2:**
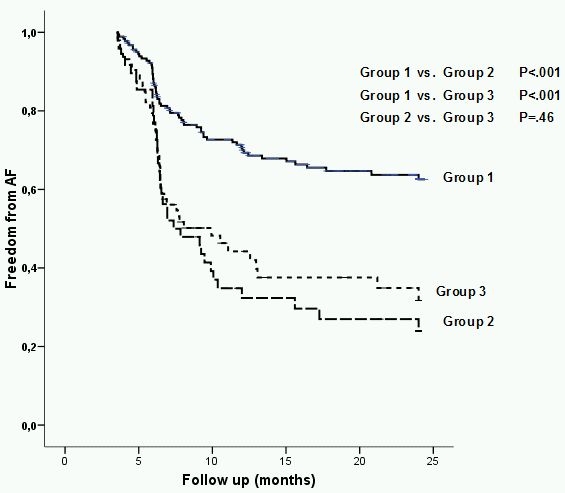
Outcome after catheter ablation in different AF groups. Group 1-paroxysmal AF with AF burden < 500 hours/3 months; Group 2-paroxysmal AF with AF burden ≥ 500 hours/3 months; Group 3-persistent AF

Out of 320 enrolled patients, 182 (57 %) were free of documented AF recurrence during 2-year follow-up. Kaplan-Meier survival analysis has shown that dichotomized AF burden was strongly associated with outcome (Figure 2). The log-rank test demonstrated significant differences in the likelihood of AF recurrence between Group 1 (n=192), and Group 2(n=52) (χ^2^=26.11, p< .001), and Group 3 (n=76) (χ^2^=20.17, p< .001). Of note, no significant difference between Group 2 and Group 3 was found (χ^2^=2.43, p=.46).

In univariate analysis, AFB ≥ 500 hours, LALD ≥ 60 mm, and the presence of coronary artery disease were associated with the recurrence of AF (Table 5). In multivariate analysis, only AFB ≥ 500 hours/3 months and LALD ≥ 60 mm were independent predictors of AF recurrence. Neither ablation tools, nor history of AF, presence of heart disease, and other clinical parameters have been shown to be independent predictors of outcome (Table 6).

The detailed data on outcome according to the energy source used is given in the table 7. No impact of energy source was found in patients assigned to Groups 1 and 2. The patients from Groups 2 and 3 had overall reduced outcome. However, patients treated with a cryoballoon had the highest recurrence rate among patients with persistent AF (Table 7).

**Figure 3:**
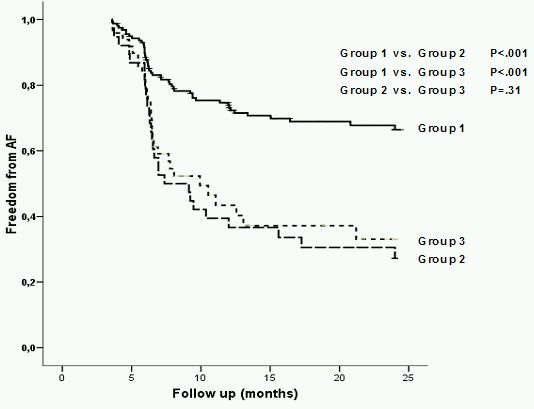

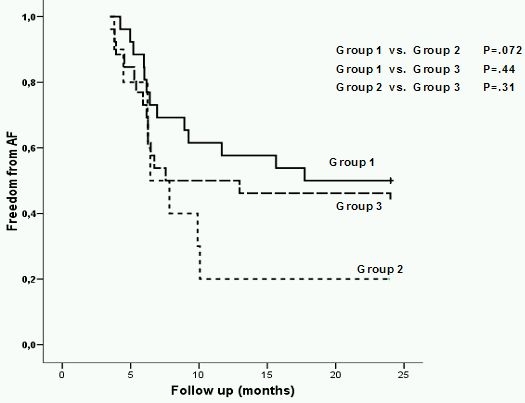
Outcome after catheter ablation patients according to LALD. a) LALD < 60 mm; b) LALD≥ 60 mm; The curves present follow AF groups: Group 1 -paroxysmal AF with AF burden < 500 hours/3 months; Group 2 -paroxysmal AF with AF burden ≥ 500 hours/3 months; Group 3 -persistent AF; a) Best outcome shows Group 1, whereas no differences could be found between Group 2 and Group 3. b)  No differences in outcome could be found

The subgroup analysis showed significant differences in outcome between Group 1 and other AF groups in patients with LALD< 60 mm. (Figure 3.a). In contrast, no significant differences could be found between all AF groups in patients with LALD ≥ 60 mm (Figure 3.b).

In 27 patients (8.4 %) left atrial tachycardia (LAT) was observed during follow-up. In 19 of them (70 %) LAT was transient and in 8 patients (30 %) additional ablation was needed.

## Discussion

The main finding of this study is that symptomatic AF burden can be used for prediction of outcome patients with PAF. The quantitative evaluation of symptomatic AFB based on the patient diary prior to procedure allowed discrimination between patients in low or high risk groups and may be useful for the choice of the appropriate ablation strategy. We found that symptomatic AFB before ablation had significantly better outcome, whereas no difference was found between patients with PAF and AFB ≥ 500 hours within the last 3 months prior to PVI and patients with persistent AF. Poor outcome after PVI in patients with persistent AF and in up to 40% patients with PAF was observed in several previous studies [1-6]. On the other hand, up to 25% of patients with initially diagnosed PAF progressed to persistent AF [14]. However, patients with frequent and/or prolonged episodes of AF who may be referred as "progressing to persistent AF", have not been studied as a separate entity. Similar recurrence rates in patients with persistent AF and patients with PAF and high AFB Figure 3.a500 h/ 3 months allow the later group to be considered as being in progression to persistent AF.

Important is the fact that patients with low AFB have better outcome irrespective of the ablation strategy. For example, these patients may be considered for PVI with the cryoballoon. This ablation technique has been shown to be effective for the majority of patients with PAF and free from such severe complications as esophageal fistula and PV stenosis as well [15]. Consequently, patients with increased AFB should be treated as patients with persistent AF with PVI and extended left atrial ablation with radiofrequency energy [16,17].

The association between the left atrial size and high recurrence rates after ablation was described in several previous studies [6-8,18]. Although left atrial enlargement may frequently occur asymmetrically [12,19], only short diameter of left atrium has been usually evaluated [6-8]. The left atrial volume seems to be a more accurate parameter of left atrial size than left atrial diameter [12,19-21]. However, there is no consensus regarding the standard method for assessment of the left atrial volume based on 2-D measurement [19]; the 3-D echo investigation has recently become available. Using a four-chamber view, we observed many cases of asymmetric enlargement of the left atrium, so that the short diameter was below the upper limit of normal and the long diameter was considerably increased (Figure 1). The cut-off point for long diameter which allowed high and low risk patients to be discriminated between was 60 mm. Even patients with low AFB prior to the procedure but increased long diameter of left atrium were more likely to have AF recurrence after single PVI (Figure 3.b).

## Conclusion

Our results suggest that patients with PAF and very high AFB must be considered as being in progression to persistent AF and accordingly treated. The patients with low AFB and non-dilated left atrium have a high probability to be in sinus rhythm after single PVI. 

## Study limitation

The following limitations of the study should be acknowledged. We could not identify all episodes of AF, particularly asymptomatic, because no monitoring was performed before ablation. We instead relied on patient self-reports. Furthermore, we only assessed symptomatic burden during a limited period of 3 months before ablation. If an implantable loop-recorder was used, it would be possible to include potential asymptomatic AF burden into analysis. The last limitation we have to mention is  follow up time of 2 years.

## Figures and Tables

**Table 1:**
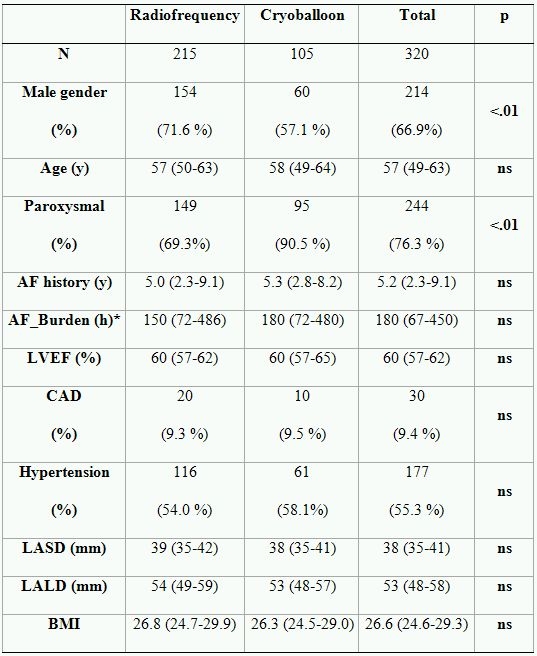
Baseline characteristics

Continuous data are given as median (IQR). * AF burden was defined as total duration of symptomatic AF episodes in patients with paroxysmal AF within 3 months prior to ablation.  LASD-Left atrial short diameter; LALD-Left atrial long diameter

**Table 2:**
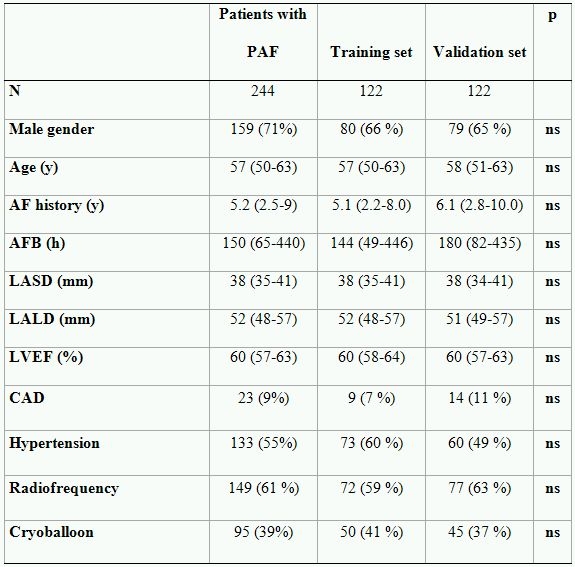
Characteristics of the training and validating set

CAD- Coronary artery disease

**Table 3:**
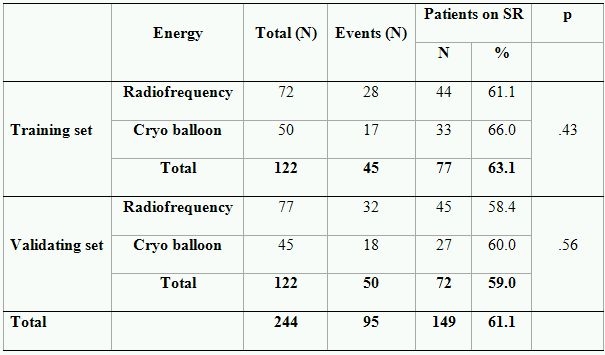
Effect of ablation energy on outcome in patients with paroxysmal atrial fibrillation

**Table 4:**
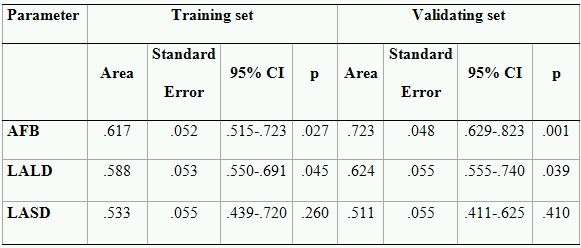
ROC curve analysis in training and validation set

**Table 5:**
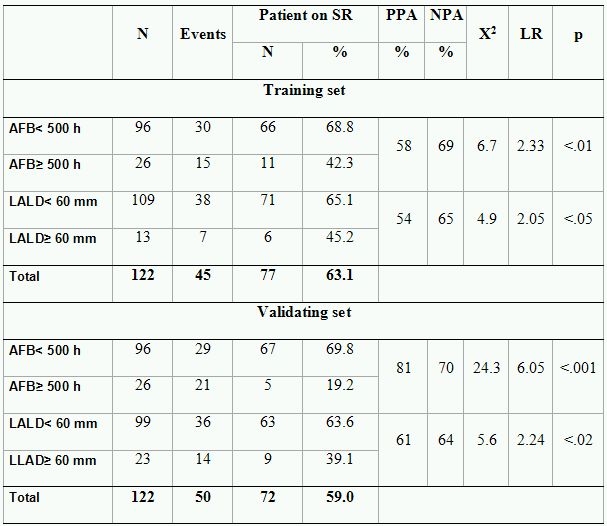
Predictive accuracy in the training and validation set

PPA- Positive predictive accuracy; NPA-Negative predictive accuracy; LR- Maximum likelihood ratio

**Table 6:**
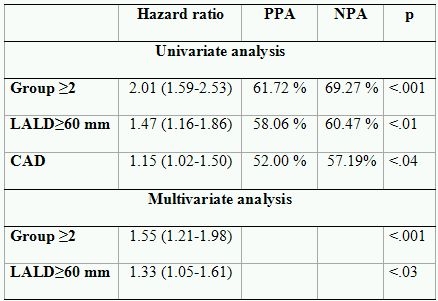
Association of clinical variables with outcome

Group ≥2- Patients with PAF and AFB≥ 500 h and patients with persistent AF

**Table 7:**
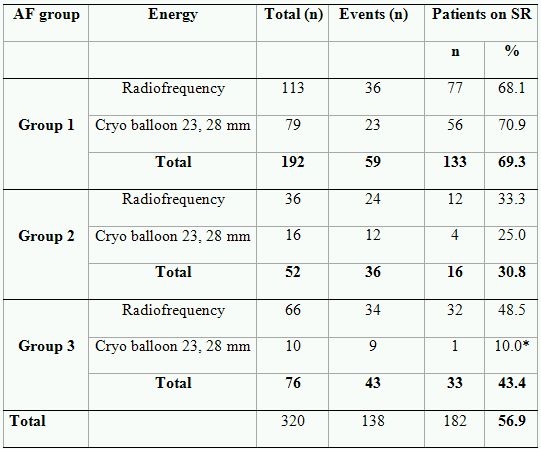
Outcome after the procedure according to energy source

* There is significant difference in outcome within group (p< .01).
